# The Time for a Consensus Regarding the Coeliac Disease Diagnosis in Adults has come: Balancing the Pros and Cons of Omitting the Biopsy

**DOI:** 10.37825/2239-9747.1027

**Published:** 2021-12-23

**Authors:** Carolina Ciacci, Julio C. Bao

**Affiliations:** a Department of Medicine, Surgery, Dentistry, Scuola Medica Salernitana, Italy; b Dr. C. Bonorino Udaondo Gastroenterology Hospital, Buenos Aires, Italy; c Research Institute, Universidad del Salvador, Buenos Aires, Italy

**Keywords:** Coeliac disease, Histology, Biopsy, Consensus, Diagnosis

## Abstract

How balancing the pros and cons of omitting the biopsy in diagnosing CeD in adults?

*To the Editor*,

Coeliac disease (CeD) is an immunomediated chronic disease that develops in genetically susceptible individuals after consuming gluten-containing cereals. The epidemiology of CeD reveals that it is a common food intolerance, affecting about 1% of the global population with a few exceptions [1]. The blood levels of IgA type anti-tissue transglutaminase (tTG) antibodies and the histology of perendoscopic intestinal biopsies showing mucosal damage are considered the “gold standard” to make the diagnosis [2]. Since its introduction in the mid-1950s, duodenal biopsy has been essential [3,4]. Upper GI endoscopy became the preferred method for obtaining duodenal biopsies in the 1980s [5]. Aseminal study published in 1996 investigated, for the first time, the possibility of diagnosing CeD in adults solely based on specific serology (anti-endomysial: 100% positive predictive value and 70% sensitivity) [6]. As a result, several studies with a similar goal of investigating adult and children patients were conducted. Based on all of the previous studies, the ESPGHAN established in 2012 that high levels of tTG IgA (x10) accurately predicted the presence of atrophic intestinal mucosa in symptomatic patients. Further ESPGHAN-funded research confirmed that the serological strategy could replace biopsy in children, even in asymptomatic cases [7]. As a result, a new ESPGHAN consensus confirmed that in children omitting the biopsy in the presence of a high anti-tTG IgA titer is a safe and recommended practice in diagnosing CeD [7].

There is an exciting debate among CeD experts about the possibility of omitting the biopsyin adults when the titer of anti-tTGis high. The topic reasonably divides the CeD experts for several reasons.

First, a diagnosis of CeD is forever. The idea of confirming by histology the serology findings is reassuring. However, the same is for children and adults, and for children, the no-biopsy strategy is now the standard of care.

Secondly, there is a reasonable worry to miss the diagnosis of diseases other than CeD. In the suspicion of CeD for digestive symptoms or iron deficiency, the upper endoscopy might unveil other diseases such as peptic ulcer and gastric cancer, besides obtaining the intestinal biopsy. However, data from the literature are reassuring as in a multicenter study, coincident significant UGI endoscopic findings appear to be rare in adult CeD [8].

Another con toward the biopsy is that CeD is considered a precancerous disease, and adults may receive a late diagnosis of CeD. The long-time gluten exposure and the presence of symptoms increase the frequency of the most alarming complication of CeD, the intestinal T-cell lymphoma [9].

Nevertheless, lymphoma diagnosis is relatively infrequent at the endoscopy, although the diagnosis of lymphoma and CeD might be almost simultaneous [10].

Finally, the histology allows the diagnosis of potential CeD, i.e., positive anti tTG Ig A and absence of mucosa damage. The potential CeD remains a problem to solve as a non-neglectable percentage of potential CeD patients may never develop mucosal damage [11].

Which are the pros of omitting the biopsy? Indeed, the presence of high titer anti-tTG IgA in adults shows the same accuracy as in children in predicting intestinal mucosa atrophy. Endoscopy is an unpleasant and expensive procedure. Histology is often operator-dependent; it also resents good sampling and orientations of the specimen.

The question now is to balance the pros and cons of omitting the biopsy in diagnosing CeD in adults.

The right time has come to put together all the scientific evidence and ask a group of experts to reach a consensus on the issue. The consensus would possibly select the appropriate condition(s) for which the omission of the endoscopy would be safe regarding missing other diseases and granting accuracy for the diagnosis of CeD.
